# Supplementary material for: Causal Relationship between Aging and Anorexia Nervosa: A White-Matter-Microstructure-Mediated Mendelian Randomization Analysis
Source: Biomedicines. 2024 Aug 16;12(8):1874. doi: 10.3390/biomedicines12081874 (PMC11351342; doi:10.3390/biomedicines12081874)
Supplement: Supplementary file 1 [file biomedicines-12-01874-s001.zip › Supplementary Figure/Figure S4/Singe SNP plot of MR analysis for TL to ubm-b-1581.pdf]

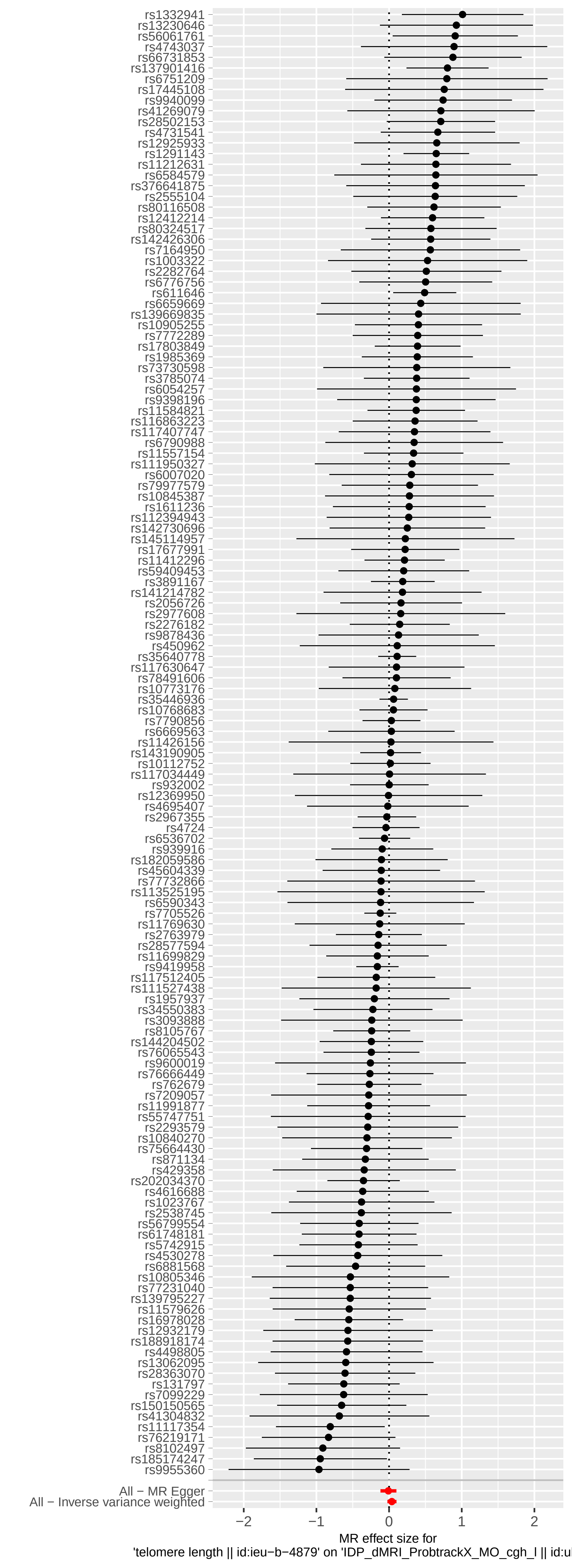
rs1332941
rs13230646
rs56061761
rs4743037
rs66731853
rs137901416
rs6751209
rs17445108
rs9940099
rs41269079
rs28502153
rs4731541
rs12925933
rs1291143
rs11212631
rs6584579
rs376641875
rs2555104
rs80116508
rs12412214
rs80324517
rs142426306
rs7164950
rs1003322
rs2282764
rs6776756
rs611646
rs6659669
rs139669835
rs10905255
rs7772289
rs17803849
rs1985369
rs73730598
rs3785074
rs6054257
rs9398196
rs11584821
rs116863223
rs117407747
rs6790988
rs11557154
rs111950327
rs6007020
rs79977579
rs10845387
rs1611236
rs112394943
rs142730696
rs145114957
rs17677991
rs11412296
rs59409453
rs3891167
rs141214782
rs2056726
rs2977608
rs2276182
rs9878436
rs450962
rs35640778
rs117630647
rs78491606
rs10773176
rs35446936
rs10768683
rs7790856
rs6669563
rs11426156
rs143190905
rs10112752
rs117034449
rs932002
rs12369950
rs4695407
rs2967355
rs4724
rs6536702
rs939916
rs182059586
rs45604339
rs77732866
rs113525195
rs6590343
rs7705526
rs11769630
rs2763979
rs28577594
rs11699829
rs9419958
rs117512405
rs111527438
rs1957937
rs34550383
rs3093888
rs8105767
rs144204502
rs76065543
rs9600019
rs76666449
rs762679
rs7209057
rs11991877
rs55747751
rs2293579
rs10840270
rs75664430
rs871134
rs429358
rs202034370
rs4616688
rs1023767
rs2538745
rs56799554
rs61748181
rs5742915
rs4530278
rs6881568
rs10805346
rs77231040
rs139795227
rs11579626
rs16978028
rs12932179
rs188918174
rs4498805
rs13062095
rs28363070
rs131797
rs7099229
rs150150565
rs41304832
rs11117354
rs76219171
rs8102497
rs185174247
rs9955360
All − MR Egger
All − Inverse variance weighted
−2
−1
0
1
2
MR effect size for
'telomere length || id:ieu−b−4879' on 'IDP_dMRI_ProbtrackX_MO_cgh_l || id:u
